# Multi-criteria healthcare waste disposal location selection based on Fermatean fuzzy WASPAS method

**DOI:** 10.1007/s40747-021-00407-9

**Published:** 2021-06-18

**Authors:** Arunodaya Raj Mishra, Pratibha Rani

**Affiliations:** 1 Department of Mathematics, Government College Jaitwara, Satna, MP 485221 India; 2 Department of Mathematics, National Institute of Technology Warangal, Telangana, 506004 India

**Keywords:** Fermatean fuzzy sets, Entropy, Score function, Healthcare waste disposal location, WASPAS

## Abstract

Medical services inevitably generate healthcare waste (HCW) that may become hazardous to healthcare staffs, patients, the population, and the atmosphere. In most of the developing countries, HCW disposal management has become one of the fastest-growing challenges for urban municipalities and healthcare providers. Determining the location for HCW disposal centers is a relatively complex process due to the involvement of various alternatives, criteria, and strict government guidelines about the disposal of HCW. The objective of the paper is to introduce the WASPAS (weighted aggregated sum product assessment) method with Fermatean fuzzy sets (FFSs) for the HCW disposal location selection problem. This method combines the score function, entropy measure, and classical WASPAS approach within FFSs context. Next, a combined procedure using entropy and score function is proposed to estimate the criteria weights. To do this, a novel score function with its desirable properties and some entropy measures are introduced under the FFSs context. Further, an illustrative case study of the HCW disposal location selection problem on FFSs is established, which evidences the practicality and efficacy of the developed approach. Comparative discussion and sensitivity analysis are made to monitor the permanence of the introduced framework. The final results approve that the proposed methodology can effectively handle the ambiguity and inaccuracy in the decision-making procedure of HCW disposal location selection.

## Introduction

During the last 5 decades, the population has increased very fast all over the globe, mainly in developing nations. This rapid growth has generated various concerns which have a severe impact on humans and animals’ wellbeing. One of those concerns is the disposal of large amounts of healthcare waste (HCW) generated from medical centers, laboratories, and hospitals. The World Health Organization (WHO) describes HCW as “wastes generated by the healthcare activities includes a broad range of materials, used syringes, blooded cottons, bandages, scalpels, body parts, chemicals, cytotoxic, and radioactive components”. According to the WHO, approximately 85% of HCW is non-hazardous, while the other 15% is hazardous that maybe contagious, toxic or radioactive. If these wastes are not properly handled or disposed, then these 15% of HCW pose different types of ecological and health risks [11, 24]. As a matter of fact, providing an environmental-friendly and proper HCW management system is one of the major concerns for health care organizations [39, 49]. HCW management systems offer services for an assortment of waste produced by the healthcare services, assess transit modes and routes for transport waste to treatment plants, and help to choose the treatment alternative and the disposal location. Due to rapid population growth and a number of increasing healthcare services, the quantity of HCW composed for treatment and disposal is growing quickly (IndiaStat [24], as a consequence, the selection of suitable HCW disposal location is an important concern to establish a facility for daily storage, treatment and disposal of HCW [12].

The location selection for HCW disposal is a strategic critical problem faced by the healthcare specialists and municipality. In the existing studies, the facility location problems have been discussed for supply chain management which considers the concerning assessment of enterprises’ production, warehouse, and distribution center location [3, 16, 29]. Some more problems comprise the decision related to the waste storage bins in medical/healthcare centers, location of medical/healthcare centers, and others [6, 29, 38]. In a study, Ertugrul and Karakasoglu [16] discussed multi-criteria facility location problem depend on certain criteria namely availability of labour. Nonetheless, a proper HCW disposal location cannot be restricted to certain basic criteria but also considers all the dimensions of sustainability, i.e., social, economic, and environmental aspects. Thus, the selection of most suitable HCW disposal location among a set of alternative locations is a complex multi-criteria decision-making (MCDM) process due to the presence of multiple qualitative and quantitative criteria from sustainable perspectives [56].

Uncertainty is frequently occurred in the HCW disposal location process due to the presence of multiple constraints, lack of knowledge, vague human mind, and inconsistency of the problem. The theory of fuzzy sets (FSs) [57] has effectively been implemented in multiple real-life MCDM problems and evidenced its dominant ability to handle the imprecise and uncertain information. As an improvement of FSs, the notion of Fermatean fuzzy sets (FFSs) [44] has been proven as a more superior tool to model the imprecise and uncertain information that arises in real-world applications. Corresponding to its unique advantages, this study focuses on the FFSs environment. Therefore, the goal and motivation of the work are to display how the entropy and improved score function can be used for the evaluation of the degree of uncertainty and comparability of Fermatean fuzzy numbers (FFNs), respectively. As far as we know, there is no work in the literature regarding a MCDM methodology based on the combination of entropy measure, score function and WASPAS (weighted aggregated sum product assessment) model under FFS environment, named as Fermatean fuzzy-WASPAS (FF-WASPAS). Further, this method is implemented in the assessment of the HCW disposal location selection problem. Consequently, the key contributions of the paper are described as.

- Novel Fermatean fuzzy-WASPAS (FF-WASPAS) method based on score function and entropy measure is developed for solving complex MCDM problems.
- An improved score function and entropy measure for FFSs are developed with their elegant properties. And, further employed to assess the criteria weights.
- To display the feasibility and effectiveness of the developed methodology, a practical case study of HCW disposal location selection is presented in the FFSs context.
- Comparative study is conferred to exemplify the validity and stability of the introduced framework.

Hence, the present manuscript has the following key research implications: (1) the first aim is to endure an ample survey on HCW management procedures; (2) the second aim is to choose and assess the most appropriate location for HCW disposal under uncertainty using an integrated framework; (3) third aim is to develop a new Fermatean fuzzy entropy and improved score function-based framework for handling imprecise data in MCDM problems and (4) lastly, a real application of HCW disposal location selection in Uttarakhand, India is taken with sustainable perspectives.

The rest part of this study is planned as follows: “Earlier works” discusses systematic reviews related to the present study. “Preliminaries” discusses the basic concepts of FFSs. “Improved score function and entropy measure within FFSs” presents a novel Fermatean fuzzy score function and some new entropy measures under FFSs environment. “Proposed FF-WASPAS method for MCDM problems” proposes a novel Fermatean fuzzy WASPAS model based on score function and entropy measure with FFSs. “Case study: healthcare waste disposal location (HCWDL) selection” offers a case study of HCW disposal location selection with FFSs setting. Furthermore, a comparison with extant approaches is made to show the feasibility and stability of the obtained outcomes. Lastly, “Conclusions” confers the conclusion of the study and future research direction.

## Earlier works

The current section discusses the appropriate literature on the FFSs, WASPAS approach, location selection, and waste disposal processes.

### Fermatean fuzzy sets

In 1986, the theory of intuitionistic fuzzy set (IFS) was originated by Atanassov [8], which is portrayed by the membership degree (MemD) and non-membership degree (NMemD), and fulfills a constraint that the addition of MemD and NMemD is restricted to one. Based on its unique advantages, IFS has appeared as one of the valuable means for characterizing uncertainty and vagueness of real-life problems [5, 22, 31]. Further, Yager [55] established the concept of Pythagorean fuzzy set (PFS), an extension of IFS, expressed by the MemD and NMemD, and fulfills a condition that the square addition of MemD and NMemD is restricted to one. As a result, PFSs are more advantageous than IFSs for handling the uncertainty and imprecise information obtained in realistic problems. Recently, numerous studies have been presented to explore the PFSs from different perspectives [43, 48, 51].

Though, in numerous realistic decision-making problems, there may be a case in which experts present his/her opinion as (0.8, 0.7). Consequently, IFS and PFS are unable to deal with this situation because $0.8+0.7>1$ and ${0.8}^{2}+{0.7}^{2}>1$. In order to illustrate this issue, [44] introduced the idea of the Fermatean fuzzy set (FFS). The FFSs are portrayed by the MemD and NMemD such that the cube sum of MemD and NMemD is restricted to one. Accordingly, the FFSs are more influential and effective way than IFSs and PFSs in solving uncertain MCDM problems. With increasing complexity and extensive changes of environment, FFSs have grown a momentous consideration from authors. Senapati and Yager [44–46] discussed several basic operational laws, score and accuracy functions, some weighted averaging/geometric operators for FFSs and their applications in MCDM problems. Aydemir and Gunduz [9] extended the Dombi aggregation operators for FFSs. Recently, Mishra et al. [32] studied an integrated MCDM methodology by CRiteria Importance Through Intercriteria Correlation (CRITIC) and Evaluation Based on Distance from Average Solution (EDAS) approaches under FFS context. Nonetheless, no one has focused their attention on location selection for HCW disposal under FFSs context.

### WASPAS method

Recently, several MCDM models have been proposed under diverse uncertain settings. Zavadskas et al. [59] established the notion of the weighted aggregated sum product assessment (WASPAS) model, which has been extensively utilized in numerous realistic applications. It is a combination of the weighted sum model (WSM) and weighted sum model (WPM), and is more precise than these models. To cope with uncertain information of MCDM problems, the WASPAS model has been expanded under diverse fuzzy environments (see Table 1. It is observed from the existing studies that there is no study to develop the classical WASPAS to Fermatean fuzzy WASPAS (FF-WASPAS) methodology with entropy and score function under FFSs context.

**Table 1:** Comprehensive review of WASPAS method

References	Method	Application
Zavadskas et al. [58]	Single-valued neutrosophic WASPAS	Construction of alternative sites for waste incineration plant
Turskis et al. [52]	Fuzzy AHP-WASPAS	Shopping center construction site selection
Ghorabaee et al. [19]	Interval type-2 fuzzy WASPAS	Evaluation of green suppliers
Deveci et al. [13]	Interval type-2 fuzzy- WASPAS-TOPSIS	Assessment of car-sharing station
Mishra and Rani [33]	Interval-valued intuitionistic fuzzy WASPAS	Evaluation and selection of reservoir flood control management policies
Mishra et al. [34]	Hesitant fuzzy WASPAS	Green supplier selection
Schitea et al. [42]	Intuitionistic fuzzy WASPAS-COPRAS-EDAS	Hydrogen mobility roll-up site selection
Rani and Mishra [41]	q-rung orthopair WASPAS	Alternative fuel technologies assessment
Mohagheghi and Mousavi [35]	Interval-valued Pythagorean fuzzy D-WASPAS	Evaluation of sustainable project portfolios
Mardani et al. [28]	Hesitant fuzzy-SWOT-SWARA-WASPAS	Assessment of digital technologies intervention to control the COVID-19 outbreak
Rani et al. [43]	Intuitionistic fuzzy type-2 WASPAS	Physician selection
Agarwal et al. [1]	Fuzzy SWARA-WASPAS	Evaluation of humanitarian supply chain management barriers
Ali et al. [2]	Uncertain probabilistic linguistic WASPAS	Supplier selection

### Location selection and waste disposal system

Location assessment for HCW disposal and collection of disposal can be demonstrated as an interesting and vital concern for municipality and industrialists. In the literature, several scholars have focused their researches on the investigations of medical wastes, factory wastes, and solid municipal wastes. Karamouz et al. [26] utilized a framework to rank the hospitals in Ahvaz, Iran in terms of hospital waste collection. Ekmekçioğlu et al. [14] discussed an integrated framework with AHP and TOPSIS models for assessing municipal waste treatment techniques in Istanbul under FSs. Şener et al. [47] oriented the GIS and AHP-based framework to evaluate the landfill sites in Turkey. Mokhtarian et al. [36] used VIKOR model under interval-valued FSs to choose a suitable location for municipal wet waste disposal. Khan and Samadder (2015) utilized a system to assess and rank the municipal waste landfill sites from sustainable perspectives. In another study, a collective MCDM model using AHP and WASPAS models with IVNSs was recommended to rate and analyze all the locations to select the suitable one [58]. Kahraman et al. [25] developed EDAS model to evaluate and rank the potential solid waste disposal locations on IFSs. Wichapa and Khokhajaikiat [53] used AHP and goal programming model on FSs to assess the suitable infectious waste disposal location in Thailand.

Chauhan and Singh [12] discussed a combined model with TOPSIS and AHP methods to opt a most favorable HCW disposal location facility under FSs. Eskandari et al. [17] used an integrated tool to appraise the suitability of the solid waste landfill location of a southern province in Iran. In another study in Kenya, Hariz et al. [21] applied a decision mechanism including GIS and various MCDM tools namely AHP, VIKOR, and PROMETHEE models to choose a suitable HCW disposal location facility. Arıkan et al. [7] reported TOPSIS-PROMETHEE model under FSs to choose the suitable solid waste disposal locations of Istanbul, Turkey. Further, Thakur and Ramesh [50] presented a grey measure-based AHP to solve the HCW disposal system in India. Gergin et al. [18] utilized ABC process to establish the best waste disposal location in Istanbul. Yazdani et al. [56] established a rough theory-based decision support system for evaluating and choosing the most suitable location for HCW disposal. Mishra et al. [31] pioneered a cross entropy-based EDAS model to calculate the HCW disposal method under IFSs.

## Preliminaries

In the following, several important ideas associated with Fermatean fuzzy sets are given:

### Definition 3.1

In 2019, Senapati and Yager [44] defined the mathematical form of a FFS $\tilde{F }$, given as.

1 $$ \tilde{F}\, = \,\left\{ {\left. {\left\langle {z_{i} ,\,\left( {t_{{\tilde{F}}} (z_{i} ),\,f_{{\tilde{F}}} (z_{i} )} \right)} \right\rangle } \right|\,z_{i} \, \in \,\Omega } \right\}, $$

where $\Omega$ denotes the finite discourse set and $t_{{\tilde{F}}} \,:\,\Omega \, \to \,\left[ {0,\,1} \right]$ and $\nu_{{\tilde{F}}} \,:\,\Omega \, \to \,\left[ {0,\,1} \right]$ symbolize the MemD and NMemD of an element $z_{i} \in \Omega$ to the set$\tilde{F }$, respectively, under a constraint $0\, \le \,\left( {t_{{\tilde{F}}} \left( {z_{i} } \right)} \right)^{3} \, + \,\left( {f_{{\tilde{F}}} \left( {z_{i} } \right)} \right)^{3} \, \le \,1.$ For each $z_{i} \, \in \,\Omega ,$ the hesitancy degree is described as $\pi _{{\tilde{F}}} \left( {z_{i} } \right) = \sqrt[3]{{1 - t_{{\tilde{F}}}^{3} \left( {z_{i} } \right) - f_{{\tilde{F}}}^{3} \left( {z_{i} } \right)}}$. Further, the concept of Fermatean fuzzy number (FFN) is given by Senapati and Yager [44], and represented as $o = \,\left( {t_{o} ,\,f_{o} } \right)$ in which $t_{o} ,\,f_{o} \, \in \,\left[ {0,\,1} \right]$ and $0\, \le \,t_{o}^{3} \, + \,f_{o}^{3} \, \le \,1.$

In Definition 3.2, a relation between FFNs is given to compare the two FFNs as follows:

### Definition 3.2

For a FFN $o = \,\left( {t_{o} ,\,f_{o} } \right)$, Senapati and Yager [44, 45] defined the concept of score function, given as

2 $$ \widetilde{{\mathbb{S}}}\left( o \right) = \left( {t_{o} } \right)^{3} - \left( {f_{o} } \right)^{3} $$

wherein $$\widetilde{{\mathbb{S}}}\left( o \right) \in \left[ { - 1,\,1} \right].$$

Based on Eq. (2), it can easily be observed that score function $\widetilde{{\mathbb{S}}}\left( o \right)$ of the FFN ‘*o*’ is directly related to the difference between the MemD and the NMemD. The larger the score value, the higher the FFN.

Further, the accuracy function of a FFN $o = \,\left( {t_{o} ,\,f_{o} } \right)$ is defined as [44, 45]

3 $$ \tilde{\hbar }\left( o \right) = \left( {t_{o} } \right)^{3} + \left( {f_{o} } \right)^{3} ;\,\,\,\tilde{\hbar }\left( o \right) \in \left[ {0,1} \right]. $$

The bigger the accuracy function, the larger the FFN. The most significant contributions of Definition 3.2 is that it gives a procedure to compare different FFNs.

Based on Eqs. (2) and (3), a comparative scheme is presented for any two FFNs $o_{1} = \,\left( {t_{{o_{1} }} ,\,f_{{o_{1} }} } \right)$ and $o_{2} = \,\left( {t_{{o_{2} }} ,\,f_{{o_{2} }} } \right),$

(i) If $$\widetilde{{\mathbb{S}}}\left( {o_{1} } \right) > \widetilde{{\mathbb{S}}}\left( {o_{2} } \right),$$ then $$o_{1} > \,o_{2} ,$$
(ii) If $\widetilde{{\mathbb{S}}}\left( {o_{1} } \right) = \widetilde{{\mathbb{S}}}\left( {o_{2} } \right),$ then
  - If $$\tilde{\hbar }\left( {o_{1} } \right) > \tilde{\hbar }\left( {o_{2} } \right),$$ then $$o_{1} > \,o_{2} ;$$
  - If $$\tilde{\hbar }\left( {o_{1} } \right) = \tilde{\hbar }\left( {o_{2} } \right),$$ then $$o_{1} = \,o_{2} .$$

For handling the FFN better, a set of operations on FFNs is described as follows:

### Definition 3.3

For three FFNs $o = \,\left( {t_{o} ,\,f_{o} } \right),$ $o_{1} = \,\left( {t_{{o_{1} }} ,\,f_{{o_{1} }} } \right)$ and $o_{2} = \,\left( {t_{{o_{2} }} ,\,f_{{o_{2} }} } \right),$ the basic set operational laws on FFNs are defined by Senapati and Yager [44, 45].

(i) $$o^{c} \, = \left( {f_{o} ,\,t_{o} } \right),$$
(ii) $$o_{1} \, \cap \,o_{2} \, = \,\left( {\min \left\{ {t_{{o_{1} }} \,,\,t_{{o_{2} }} } \right\},\,\,\,\max \left\{ {f_{{o_{1} }} \,,\,f_{{o_{2} }} } \right\}} \right),$$
(iii) $$o_{1} \, \cup \,o_{2} \, = \,\left( {\max \left\{ {t_{{o_{1} }} \,,\,t_{{o_{2} }} } \right\},\,\,\,\min \left\{ {f_{{o_{1} }} \,,\,f_{{o_{2} }} } \right\}} \right),$$
(iv) $$ o_{1} {\mkern 1mu}  \oplus {\mkern 1mu} o_{2} {\mkern 1mu}  = {\mkern 1mu} \left( {\sqrt[3]{{t_{{o_{1} }}^{3} {\mkern 1mu}  + {\mkern 1mu} t_{{o_{2} }}^{3} {\mkern 1mu}  - {\mkern 1mu} t_{{o_{1} }}^{3} {\mkern 1mu} t_{{o_{2} }}^{3} }},{\mkern 1mu} f_{{o_{1} }} {\mkern 1mu} f_{{o_{2} }} } \right), $$
(v) $$ o_{1} {\mkern 1mu}  \otimes {\mkern 1mu} o_{2} {\mkern 1mu}  = {\mkern 1mu} \left( {t_{{o_{1} }} {\mkern 1mu} t_{{o_{2} }} ,{\mkern 1mu} \sqrt[3]{{f_{{o_{1} }}^{3} {\mkern 1mu}  + {\mkern 1mu} f_{{o_{2} }}^{3} {\mkern 1mu}  - {\mkern 1mu} f_{{o_{1} }}^{3} {\mkern 1mu} f_{{o_{2} }}^{3} }}} \right), $$
(vi) $$ \lambda {\mkern 1mu} o{\mkern 1mu}  = {\mkern 1mu} \left( {\sqrt[3]{{1 - \left( {1 - {\mkern 1mu} t_{o}^{3} } \right)^{\lambda } }},{\mkern 1mu} \left( {f_{o} } \right)^{\lambda } } \right),{\mkern 1mu} \;\lambda {\text{ > }}{\mkern 1mu} 0, $$
(vii) $$ o^{\lambda } {\mkern 1mu}  = {\mkern 1mu} \left( {\left( {t_{o} } \right)^{\lambda } ,{\mkern 1mu} \sqrt[3]{{1 - \left( {1 - {\mkern 1mu} f_{o}^{3} } \right)^{\lambda } }}} \right),\;\lambda  > {\mkern 1mu} 0. $$

## Improved score function and entropy measure within FFSs context

The current section presents a new score function to compare the FFSs suitably. Next, some new entropy measures are proposed for FFS.

### Novel Fermatean fuzzy Score function

The extant score function [44, 45] cannot precisely rank the FFNs in some special cases. To avoid the shortcoming of the existing score function given in Definition 3.2, the present section proposes a novel Fermatean fuzzy score function.

Suppose $o_{j} = \left( {t_{j} ,\,f_{j} } \right)$ be a FFN. Now, the score function for a FFN $o_{j}$ is proposed as follows:

4 $$ {\mathbb{S}}\left( {o_{j} } \right) = \frac{1}{2}\left[ {\left( {t_{j}^{3} \, - \,f_{j}^{3} \, - \ln \left( {1\, + \,\pi_{j}^{3} } \right)} \right) + 1} \right],\quad {\text{where}}\;{\mathbb{S}}\left( {o_{j} } \right)\, \in \,\left[ {0,\,1} \right]. $$

#### *Example 4.1*

Assume that $o_{1} = \,\left( {0.5,\,0.5} \right)$ and $o_{2} = \,\left( {0.4,\,0.4} \right)$ be the given FFNs. It is evident from Definition 3.2 that the existing score function given by Eq. (2) [44, 45] cannot discriminate the considered FFNs because $\widetilde{{\mathbb{S}}}\left( {o_{1} } \right) = \widetilde{{\mathbb{S}}}\left( {o_{2} } \right)\, = \,0$, whereas the improved score function given by Eq. (4) can effectively deal with such type of FFNs, therefore, we have ${\mathbb{S}}\left( {o_{1} } \right) = \,0.7202$ and ${\mathbb{S}}\left( {o_{2} } \right) = \,0.6865.$ Consequently, $o_{1} \, > \,o_{2} ,$ which proves the effectiveness of the improved score function over the extant one.

The proposed score function Eq. (4) satisfies the following results:

#### Theorem 4.1

*The score function* ${\mathbb{S}}\left( {o_{j} } \right),$ *given by Eq.* (4), *is increasing monotonically w.r.t.* $t_{j}$ *and decreasing monotonically w.r.t.* $f_{j} .$

#### *Proof*

The first partial derivative of Eq. (4) w.r.t. $t_{j}$ is given as.

$$ \frac{{\partial \,{\mathbb{S}}\left( {o_{j} } \right)}}{{\partial \,t_{j} }}\, = \,\frac{{3\,t_{j}^{2} }}{2}\left( {1\, + \,\frac{1}{{\left( {2 - t_{j}^{3} - \,f_{j}^{3} } \right)}}} \right)\,\,\, \ge \,0. $$

On the similar line, the first partial derivative of Eq. (4) w.r.t. $f_{j}$ is presented as

$$ \frac{{\partial \,{\mathbb{S}}\left( {o_{j} } \right)}}{{\partial \,f_{j} }}\, = \, - \frac{3}{2}\,f_{j}^{2} \,\left( {1 - \,\frac{1}{{\left( {2 - t_{j}^{3} - \,f_{j}^{3} } \right)}}} \right)\,\, \le \,0. $$

This completes the proof.

#### Theorem 4.2

*The score function is given in Eq.* (4) *fulfills the following:*

(p1). $${\mathbb{S}}\left( {(0,\,1)} \right) = \,0$$ *and* $${\mathbb{S}}\left( {(1,\,0)} \right) = \,1.$$

(p2). $$0 \le {\mathbb{S}}\left( {o_{j} } \right) \le \,1.$$

#### *Proof*

(p1). By means of Theorem 4.1, the score function ${\mathbb{S}}\left( o \right)$ acquires the least value ‘0’ or utmost value ‘1’, for $o_{j} = \left( {0,\,1} \right)$ or $o_{j} = \left( {1,\,0} \right)$, respectively. Thus, we obtain ${\mathbb{S}}\left( {o_{j} } \right)_{\min } = \,0$ and ${\mathbb{S}}\left( {o_{j} } \right)_{\max } = \,1.$

(p2). With the use of (p1), we can obtain $0 \le {\mathbb{S}}\left( {o_{j} } \right) \le \,1.$

#### Theorem 4.3

*For any two FFNs* $o_{1} = \left( {t_{1} ,\,f_{1} } \right)$ *and* $o_{2} = \left( {t_{2} ,\,f_{2} } \right),$ *if* $t_{1} \, > \,t_{2}$ *and* $f_{1} \, < \,f_{2} ,$ *then* ${\mathbb{S}}\left( {o_{1} } \right)\, > \,{\mathbb{S}}\left( {o_{2} } \right).$

#### *Proof*

From Theorem 4.1, it can be observed that the score function ${\mathbb{S}}\left( {o_{j} } \right);\,\,j = \,1,\,2$ monotonically increases over $t_{j}$ and monotonically decreases over $f_{j} ,$ respectively. Hence, the theorem is proved.

**Fig. 1:**
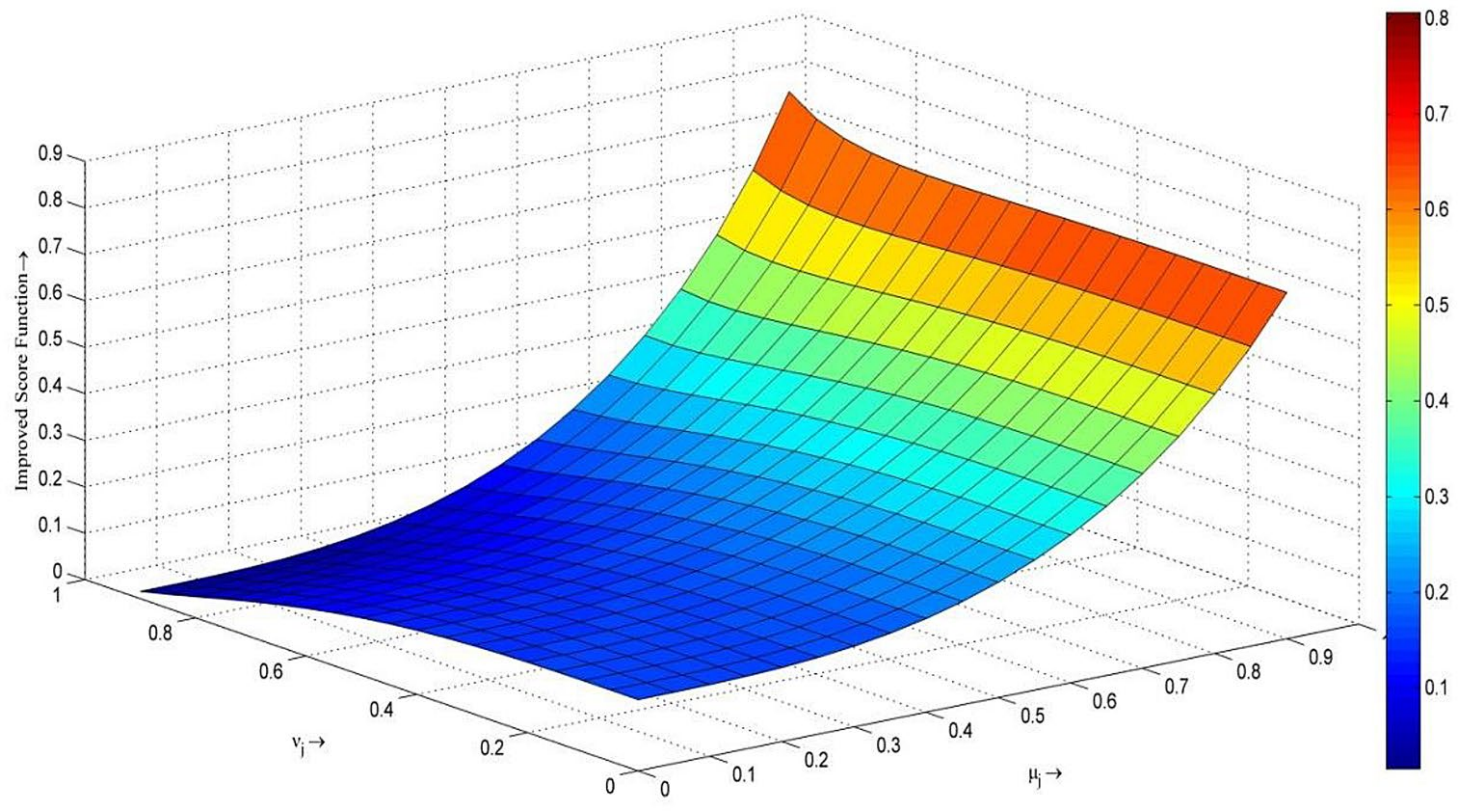
Improved score function ${\mathbb{S}}\left( {o_{j} } \right)$ with respect to parameters $\,\left( {t_{j} \,,\,f_{j} } \right)$

Moreover, Fig. 1 shows the values of score function ${\mathbb{S}}\left( {o_{j} } \right)$ over different values of $t_{j}$ and $f_{j}$. The color of each pair $\,\left( {t_{j} \,,\,f_{j} } \right)$ on the simplex demonstrates the variation on score degree of the fixed FFNs. The larger the value of $\,t_{j}$, the better the value of score function ${\mathbb{S}}\left( {o_{j} } \right)$.

### Entropy measure for FFS

In FS theory, the concept of entropy is a measure of fuzziness in a fuzzy set. The measurement of the degree of fuzziness in FSs as well as in its extended form is an important research content. A number of articles have been presented regarding the entropy measures of FS, hesitant fuzzy set, IFSs, PFS [23, 30, 43, 60], but no work is presented regarding the development of Fermatean fuzzy entropy measure.

#### Definition 4.1

A real-valued function ${\rm E}\,:\,FFS\left( \Omega \right)\, \to \,\left[ {0,\,1} \right]$ is called an entropy measure of FFSs if it fulfills the following postulates:

(P1) $$0\, \le \,\,{\rm E}\left( {\tilde{F}} \right)\,\, \le \,1,$$

(P2) ${\rm E}\left( {\tilde{F}} \right)\, = 0$ iff $\tilde{F}$ is a crisp set,

(P3) $${\rm E}\left( {\tilde{F}} \right)\, = \,1 \Leftrightarrow$$$$t_{{\tilde{F}}} \left( {z_{i} } \right) = \,f_{{\tilde{F}}} \left( {z_{i} } \right),\,\forall \,z_{i} \, \in \,\Omega \,,$$

(P4) $${\rm E}\left( {\tilde{F}} \right)\, = \,{\rm E}\left( {\tilde{F}^{c} } \right),$$

(P5) For each $z_{i} \, \in \,\,\Omega ,$ ${\rm E}\left( {\tilde{F}} \right)\, \le \,{\rm E}\left( {\tilde{G}} \right)$ if $\tilde{F}$ is less than $\tilde{G},$ i.e.,$t_{{\tilde{F}}} (z_{i} )\, \le \,t_{{\tilde{G}}} (z_{i} )\, \le \,f_{{\tilde{G}}} (z_{i} )\, \le \,f_{{\tilde{F}}} (z_{i} )$

or $$f_{{\tilde{F}}} (z_{i} )\, \le \,f_{{\tilde{G}}} (z_{i} )\, \le \,t_{{\tilde{G}}} (z_{i} )\, \le \,t_{{\tilde{F}}} (z_{i} ).$$

#### Theorem 4.4

*Let* $\tilde{F}\, \in \,FFS\left( \Omega \right).$ *Then, the some Fermatean fuzzy entropy measures are defined as*.

5 $$\begin{aligned} {\rm E}_{1} \left( {\tilde{F}} \right)\,  &= \,\frac{1}{{n\,\left( {\sqrt 2 \, - 1} \right)\,}}\,\sum\limits_{i = 1}^{n} \left\{ \sin \left( {\frac{{\pi \times \,\left( {1 + \,t_{{\tilde{F}}}^{3} \left( {z_{i} } \right) - \,f_{{\tilde{F}}}^{3} \left( {z_{i} } \right)} \right)}}{4}} \right)\right. \\ &\quad \left. + \,\sin \left( {\frac{{\pi \times \,\left( {1 - \,t_{{\tilde{F}}}^{3} \left( {z_{i} } \right)  + \,f_{{\tilde{F}}}^{3} \left( {z_{i} } \right)} \right)}}{4}} \right) - 1 \right\} , \end{aligned} $$

6 $$ \begin{aligned}{\rm E}_{2} \left( {\tilde{F}} \right)\, &= \,\frac{1}{{n\,\left( {\sqrt 2 \, - 1} \right)\,}}\,\sum\limits_{i = 1}^{n}  \left\{ \cos \left( {\frac{{\pi \times \,\left( {1 + \,t_{{\tilde{F}}}^{3} \left( {z_{i} } \right)  - \,f_{{\tilde{F}}}^{3} \left( {z_{i} } \right)} \right)}}{4}} \right)  \right. \\ &\quad  \left. + \,\cos \left( {\frac{{\pi \times \,\left( {1 - \,t_{{\tilde{F}}}^{3} \left( {z_{i} } \right) + \,f_{{\tilde{F}}}^{3} \left( {z_{i} } \right)} \right)}}{4}} \right) - 1 \right\}, \end{aligned}$$

7 $$ {\rm E}_{3} \left( {\tilde{F}} \right)\, = \,\frac{1}{n\,}\,\sum\limits_{i\, = 1}^{n} {\left[ {1 - \,\sin \left\{ {\frac{{\left( {t_{{\tilde{F}}}^{3} \left( {z_{i} } \right)\, \sim \,f_{{\tilde{F}}}^{3} \left( {z_{i} } \right)} \right)}}{{2\,\left( {1\, + \,\pi_{{\tilde{F}}}^{3} \left( {z_{i} } \right)} \right)}}} \right\}\pi } \right]} , $$

8 $$ \begin{aligned}{\rm E}_{4} \left( {\tilde{F}} \right)\, &= \,\frac{1}{2n\,}\,\sum\limits_{i\, = 1}^{n} \left[ \,\sin \left( {\frac{{t_{{\tilde{F}}}^{3} \left( {z_{i} } \right)  + \,1\, - \,f_{{\tilde{F}}}^{3} \left( {z_{i} } \right)}}{2\,}} \right)\pi \, \right. \\ &\quad \left. + \,\sin \left( {\frac{{f_{{\tilde{F}}}^{3} \left( {z_{i} } \right)\, + \,1\, - \,t_{{\tilde{F}}}^{3}  \left( {z_{i} } \right)}}{2\,}} \right)\pi  \right] , \end{aligned} $$

9 $$ {\rm E}_{5} \left( {\tilde{F}} \right)\, = \, - \frac{1}{n\,\ln 2}\,\sum\limits_{i = 1}^{n} {\left( \begin{gathered} \,t_{{\tilde{F}}}^{3} \left( {z_{i} } \right)\,\ln \left( {t_{{\tilde{F}}}^{3} \left( {z_{i} } \right)} \right) + \,f_{{\tilde{F}}}^{3} \left( {z_{i} } \right)\,\ln \left( {f_{{\tilde{F}}}^{3} \left( {z_{i} } \right)} \right) \hfill \\ \,\,\,\,\,\,\, - \left( {1 - \,\pi_{{\tilde{F}}}^{3} \left( {z_{i} } \right)} \right)\,\ln \left( {1 - \,\pi_{{\tilde{F}}}^{3} \left( {z_{i} } \right)} \right)\, - \,\pi_{{\tilde{F}}}^{3} \left( {z_{i} } \right)\,\ln 2 \hfill \\ \end{gathered} \right)} . $$

#### *Proof*

In order to verify the theorem, mapping ${\text{E}}_{i} \left( {\tilde{F}} \right)$ must hold the postulates (P1)–(P5) of Definition 4.1. As it is simple to show the postulates (P1)–(P4), so we can only verify the property (P5) for each entropy ${\rm E}_{i} \left( {\tilde{F}} \right)\,.$

(P5). Let $$\alpha \, = \,t_{{\tilde{F}}}^{3} \left( {z_{i} } \right),$$ $$\beta \, = \,f_{{\tilde{F}}}^{3} \left( {z_{i} } \right).$$ Then,

$$\phi \left( {\alpha ,\,\beta } \right) = \,\sin \left( {\frac{1 + \,\alpha \, - \,\beta }{4}\pi } \right)\, + \,\sin \left( {\frac{1 - \,\alpha \, + \,\beta }{4}\pi } \right)\, - 1,$$ where $$\alpha ,\,\beta \, \in \,\left[ {0,\,1} \right].$$

Differentiating ‘$\phi \left( {\alpha ,\,\beta } \right)$’ partially w.r.t. ‘$\alpha$’ and ‘$\beta$’, respectively, then

$$ \frac{{\partial \,\phi \left( {\alpha ,\,\beta } \right)}}{\partial \alpha } = \,\frac{\pi }{4}\,\left( {\cos \left( {\frac{1 + \,\alpha \, - \,\beta }{4}\pi } \right)\, - \,\cos \left( {\frac{1 - \,\alpha \, + \,\beta }{4}\pi } \right)} \right), $$

$$ \frac{{\partial \,\phi \left( {\alpha ,\,\beta } \right)}}{\partial \beta } = \,\frac{\pi }{4}\,\left( {\cos \left( {\frac{1 - \,\alpha \, + \,\beta }{4}\pi } \right)\, - \,\cos \left( {\frac{1 + \,\alpha \, - \,\beta }{4}\pi } \right)} \right). $$

To obtain a critical point, put $\frac{{\partial \,\phi \left( {\alpha ,\,\beta } \right)}}{\partial \alpha } = \,0$ and $\frac{{\partial \,\phi \left( {\alpha ,\,\beta } \right)}}{\partial \beta }\, = \,0$. By computing the critical value $\alpha_{c} ,$ it is found that $\alpha_{c} \, = \,b.$ Hence, if $\alpha \, \ge \,\beta ,$ then $\frac{{\partial \,\phi \left( {\alpha ,\,\beta } \right)}}{\partial \alpha } \ge \,0$ and if $\alpha \, \le \,\beta ,$ then $\frac{{\partial \,\phi \left( {\alpha ,\,\beta } \right)}}{\partial \alpha } \le \,0.$ In other words, for any $\alpha ,\,\beta \, \in \,\left[ {0,\,1} \right],$ the function $\phi$ is increasing over $\alpha$ for $\alpha \, \le \,\beta$ and decreasing when $\alpha \, \ge \,\beta .$ In the similar way, if $\alpha \, \le \,\beta ,$ then $\frac{{\partial \,\phi \left( {\alpha ,\,\beta } \right)}}{\partial \alpha } \le \,0$ and if $\alpha \, \ge \,\beta ,$ then $\frac{{\partial \,\phi \left( {\alpha ,\,\beta } \right)}}{\partial \alpha } \ge \,0.$

Suppose $$\forall \,z_{i} \, \in \,\Omega ,$$ $$t_{{\tilde{F}}} (z_{i} )\, \le \,t_{{\tilde{G}}} (z_{i} )\, \le \,f_{{\tilde{G}}} (z_{i} )\, \le \,f_{{\tilde{F}}} (z_{i} ),$$ then based on the monotonicity of $$\phi \left( {\alpha ,\,\beta } \right),$$ it is obtained as $${\rm E}_{1} \left( {\tilde{F}} \right)\, \le \,{\rm E}_{1} \left( {\tilde{G}} \right).$$ On the similar way, $$\forall \,z_{i} \, \in \,\Omega ,$$$$f_{{\tilde{F}}} (z_{i} )\, \le \,f_{{\tilde{G}}} (z_{i} )\, \le \,t_{{\tilde{G}}} (z_{i} )\, \le \,t_{{\tilde{F}}} (z_{i} ),$$ it can be observed that $${\rm E}_{1} \left( {\tilde{F}} \right)\, \le \,{\rm E}_{1} \left( {\tilde{G}} \right).$$ Thus, completes the proof.

On the similar line, Eqs. (6)–(9) can be proved.

## Proposed FF-WASPAS method for MCDM problems

**Fig. 2:**
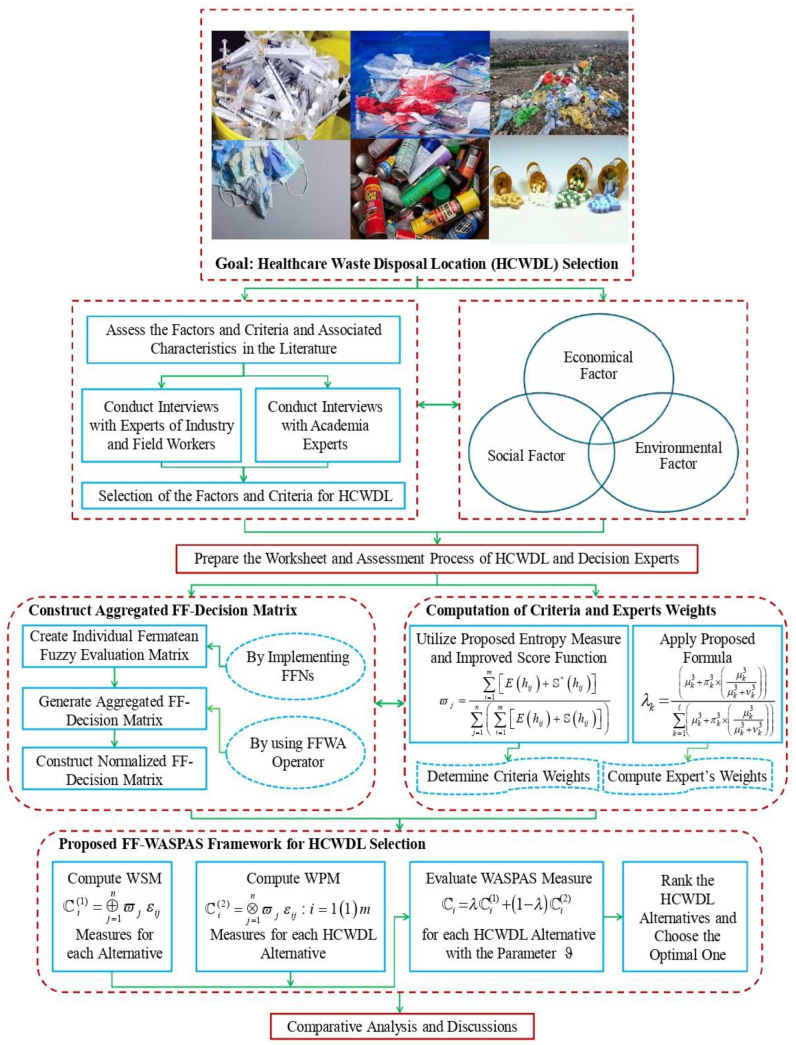
Procedural structure of proposed FF-WASPAS framework

In the present section, a collective decision-making framework is introduced with WASPAS method, entropy, and score function under FFSs environment, and named as Fermatean fuzzy WASPAS (FF-WASPAS). This methodology is proposed for solving MCDM problems with totally unidentified criteria and decision makers’ (DMs’) weights. The detailed explanation of the FF-WASPAS framework is portrayed as below (see Fig. 2):

*Step 1* Generate the FF-decision matrix (FF-DM).

Assume that there is a set of ‘*m*’ alternatives $\,\left\{ {L_{1} ,\,L_{2} ,\, \ldots ,\,L_{m} } \right\}$ and a set of ‘*n*’ attributes/criteria $\,\left\{ {V_{1} ,\,V_{2} ,\, \ldots ,\,V_{n} } \right\}$. A group of DMs $\,\left\{ {\mho_{1} ,\,\mho_{2} , \ldots ,\mho_{l} } \right\}$ is created to illustrate their views for each alternative $L_{i}$ about the attribute $V_{j}$ in the terms of FFNs. Consider that each expert $\mho_{k}$ provides his/her assessment information in terms of FF-DM $N^{(k)} = \left( {h_{ij}^{(k)} } \right)_{m \times n} .$

*Step 2* Appraise the DMs’ weights.

Let $\Gamma_{k} = \left( {t_{k} ,\,f_{k}  } \right)$ be a FFN corresponding to the linguistic variable (LV) assigned for the relative importance rating of the expert $\mho_{k} .$ Now, weight of the *k*th expert is calculated as follows [10]:

10 $$ \psi_{k} \, = \,\tfrac{{\left( {t_{k}^{3} \, + \,\pi_{k}^{3} \, \times \,\left( {\frac{{t_{k}^{3} }}{{t_{k}^{3} \, + \,f_{k}^{3} }}} \right)} \right)}}{{\sum\limits_{k\, = \,1}^{l} {\left( {t_{k}^{3} \, + \,\pi_{k}^{3} \, \times \,\left( {\frac{{t_{k}^{3} }}{{t_{k}^{3} \, + \,f_{k}^{3} }}} \right)} \right)} }},\quad {\text{such}}\;{\text{that}}\;\psi_{k} \, \ge \,0\;{\text{and}}\;\sum\nolimits_{k = 1}^{l} {\psi_{k} } = \,1. $$

*Step 3* Aggregation of the individual decision matrix.

In order to construct aggregated FF-DM (AFF-DM) $M = \left( {h_{ij} } \right)_{m\, \times \,n} ,$ the individual experts’ decisions are required to be merged into a collective decision with the help of FF-weighted averaging operator [44], where

11 $$ \begin{aligned} h_{ij} &= {\text{FFWA}}_{\psi } \,\left( {h_{ij}^{(1)} ,\,h_{ij}^{(2)} , \ldots ,h_{ij}^{(l)} } \right) \\ &= \left( {\sqrt[3]{{1 - \prod\limits_{k = 1}^{l} {\left( {1 - \left( {t_{ij}^{\left( k \right)} } \right)^{3} } \right)^{{\psi_{k} }} \,} }}\prod\limits_{k = 1}^{l} {\left( {f_{ij}^{\left( k \right)} } \right)^{{\psi_{k} }} } } \right). \end{aligned}$$

*Step 4* Compute the weights of the attributes.

To estimate the attribute weights, a novel procedure is initiated with entropy and score function under FFSs context. Let $\varpi = \left( {\varpi_{1} ,\,\varpi_{2} , \ldots ,\,\varpi_{n} } \right)^{T} ,$ where $\,\varpi_{j} \in \left[ {0,\,\,1} \right]$ and $\sum\nolimits_{j = 1}^{n} {\varpi_{j} = 1} ,$ be the weight vector of the attribute set. Then, we utilize the given procedure as follows:

12 $$ \varpi_{j} = \frac{{\sum\nolimits_{i = 1}^{m} {\left[ {{\rm E}\left( {h_{ij} } \right) + {\mathbb{S}}\left( {h_{ij} } \right)} \right]} }}{{\sum\nolimits_{j = 1}^{n} {\left( {\sum\limits_{i = 1}^{m} {\left[ {E\left( {h_{ij} } \right) + {\mathbb{S}}\left( {h_{ij} } \right)} \right]} } \right)} }},\,\,\,\forall j. $$

*Step 5* Generate the normalized aggregated FF-decision matrix (NAFF-DM).

In this MCDM procedure, the NAFF-DM ${\mathbb{N}} = \left( {\varepsilon_{ij} } \right)_{m\, \times \,n}$ from AFF-DM $M = \left( {h_{ij} } \right)_{m \times n}$ is calculated, where

13 $$ \varepsilon_{ij} = \left( {\tilde{t}_{ij} ,\tilde{f}_{ij} } \right) = \left\{ \begin{gathered} h_{ij} = \left( {t_{ij} ,f_{ij} } \right),\,\,\,\,\,\,\,\,\,\,\,\,\,\,\,\,j \in \,V_{b} , \hfill \\ \left( {h_{ij} } \right)^{c} = \left( {f_{ij} ,t_{ij} } \right),\,\,\,\,\,\,\,\,\,j \in V_{n} , \hfill \\ \end{gathered} \right. $$

where $V_{b}$ and $V_{n}$ symbolize the benefit and cost-type attributes, respectively.

*Step 6* Estimate the measure of weighted sum model (WSM) ${\mathbb{C}}_{i}^{(1)}$ for each alternative as follows:

14 $$ {\mathbb{C}}_{i}^{(1)} \, = \,\mathop \oplus \limits_{j = 1}^{n} \varpi_{j} \,\varepsilon_{ij} . $$

*Step 7* Calculated the measure of weighted product model (WPM)${\mathbb{C}}_{i}^{(2)}$ for each alternative as follows:

15 $$ {\mathbb{C}}_{i}^{(2)} \, = \,\mathop \oplus \limits_{j = 1}^{n} \varpi_{j} \,\varepsilon_{ij} . $$

*Step 8* Assess the combined measure of the WASPAS procedure for each alternative as

16 $$ {\mathbb{C}}_{i} \, = \,\lambda \,{\mathbb{C}}_{i}^{(1)} \, + \,\left( {1 - \,\lambda } \right)\,{\mathbb{C}}_{i}^{(2)} , $$

where ‘$\lambda$’ signifies the coefficient of decision mechanism, where $\lambda \in \left[ {0,\,\,1} \right]$ (when $\lambda = 0$ and $\lambda = 1,$ WASPAS is altered into the WPM and the WSM, respectively).

*Step 9* According to the values of ${\mathbb{C}}_{i} ,$ rank the given alternatives.

*Step 10* End.

## Case study: healthcare waste disposal location (HCWDL) selection

In various states and cities of India, healthcare management (HCM) is a trendy and key concern. Uttarakhand is a state of India with the diversity of cultures and traditions. The medical organization in India is categorized into public and private organizations, same as other countries. Over the past decades, the development of private healthcare centers is rapidly growing in India. Consequently, hospitals same as other organizations (industries, hotels, universities, and so on) produce pollution and a huge amount of HCWs. As the nature of HCW is hazardous and infectious for the society and the atmosphere, thus, the careful disposal of this waste becomes a necessary job for waste disposal organizations. Subsequently, finding a suitable place or location for the disposal of HCW is also an essential job for the healthcare organizations [56].

The developed methodology is utilized into three main hospitals of Uttarakhand, India namely, Rishikesh AIIMS, Government Hospital, Nirmal Ashram Hospital (NAH) to evaluate an appropriate disposal location for their HCW from sustainable perspectives. To do this, a real case study is required to reveal the practicality of the developed method. The waste management procedures of each service performed by the healthcare centers were cautiously monitored and collected the information related to management, segregation, collection, storage, and disposal of HCWs were acquired. The total waste bag produce was obtained over the half year duration from September to February of the year 2018–19. Average weights of each waste bag (color coding) from the considered hospitals are also recorded. The workers collect HCWs from these healthcare centers and safely dispose of them. Though, due to the enlargement of the healthcare center and the continuously growth in the number of patients, the administration is supposed to construct disposal location with advanced facilities. The objective of this study is to introduce a Fermatean fuzzy decision-making methodology to establish a HCWDL and reassure the healthcare executives for a careful disposal.

The process to identify the evaluation attribute was embarked on the basis of experts’ opinions, literature review, and academicians’ reports. Then, a list of attribute/criteria was identified from the relevant literature. Table 2 presents a list of ten criteria classified into three categories: environmental, economic, and social. Among the mentioned criteria, *V*_1_, *V*_3_, and *V*_6_ are non-beneficial type criteria, and rest all are of the beneficial type. Next, a group of experts is made to establish a HCW disposal center. To do this, they have conferred with the state and municipal authorities. Further, five locations in Uttarakhand are taken: ESI dispensary Jaspur, Bharat Oil and Waste Management Ltd (BOWML) Roorkee, District Hospital Bageshwar, Waste Warriors Swachhata Kendra (WWSK) Dehradun, Globe Hospital & Pharmaceutical Research Center (GHPRC) Rudrapur. The above-mentioned locations are considered as location alternatives (*L*_1_, *L*_2_, *L*_3_, *L*_4_, *L*_5_) for HCW disposal. In the evaluation process of HCWDL selection, each DM has utilized their knowledge about the considered criteria.

**Table 2:** Factors used in HCWDL assessment

Factors	Criteria	Type	References
Environmental	*V*_1_: Potential risk of intrusion and emission	Cost	Yazdani et al. [56]
	*V*_2_: Distance to the urban and city infrastructure and society	Benefit	Gorsevsky et al. [20], Zavadskas et al. [58], Yazdani et al. [56]
	*V*_3_: Distance to a complex of waste sorting	Cost	Gorsevski et al. [20], Zavadskas et al. [58], Yazdani et al. [56]
	*V*_4_: Geographic and geologic circumstances	Benefit	Ekmekçioğlu et al. [14], Gorsevski et al. [20], Yazdani et al. [56]
	*V*_5_: The prevailing environmental friendly services (air, water, energy, and electricity supply)	Benefit	Gorsevski et al. [20], Yazdani et al. [56]
Economic	*V*_6_: Land price (in m^2^) and other costs (transportation and maintenance) in the specific zone	Cost	Ekmekçioğlu et al. [14], Gorsevski et al. [20], Zavadskas et al. [58], Rakas et al. [40], Nema and Gupta [37], Chauhan and Singh [12], Arıkan et al. [7], Yazdani et al. [56]
	*V*_7_: Possibility of future development	Benefit	Gorsevski et al. [20], Yazdani et al. [56]
Social	*V*_8_: Availability of employees	Benefit	Yazdani et al. [56]
	*V*_9_: Sensitivity towards environment, local and territorial rules or protocols	Benefit	Alumur and Kara [4], Erkut et al. [15], Chauhan and Singh [12], Yazdani et al. [56]
	*V*_10_: Level of satisfaction among residents to the location selection	Benefit	Yazdani et al. [56]

*Step 1* Here, consider the rating of DMs are given as {(0.80, 0.50, 0.7133), (0.70, 0.60, 0.7612), (0.75, 0.55, 0.7440)} in terms of FFNs. Next, the FF-DM is expressed by three DMs as $N^{(k)} = \left( {h_{ij}^{(k)} } \right),\,\,k = 1,2,3$ (see Table 3).

**Table 3:** Evaluation ratings of competitive HCWDL selection

	*L*_1_	*L*_2_	*L*_3_	*L*_4_	*L*_5_
*V*_1_	$\mho_{1}$: (0.40, 0.70) $\mho_{2}$: (0.45, 0.60) $\mho_{3}$: (0.50, 0.72)	$\mho_{1}$: (0.30, 0.75) $\mho_{2}$: (0.35, 0.75) $\mho_{3}$: (0.40, 0.80)	$\mho_{1}$: (0.40, 0.65) $\mho_{2}$: (0.50, 0.70) $\mho_{3}$: (0.55, 0.72)	$\mho_{1}$: (0.30, 0.75) $\mho_{2}$: (0.50, 0.70) $\mho_{3}$: (0.45, 0.65)	$\mho_{1}$: (0.58, 0.70) $\mho_{2}$: (0.50, 0.75) $\mho_{3}$: (0.52, 0.72)
*V*_2_	$\mho_{1}$: (0.55, 0.70) $\mho_{2}$: (0.65, 0.69) $\mho_{3}$: (0.50, 0.72)	$\mho_{1}$: (0.65, 0.50) $\mho_{2}$: (0.70, 0.58) $\mho_{3}$: (0.68, 0.55)	$\mho_{1}$: (0.65, 0.58) $\mho_{2}$: (0.60, 0.52) $\mho_{3}$: (0.62, 0.54)	$\mho_{1}$: (0.58, 0.55) $\mho_{2}$: (0.60, 0.50) $\mho_{3}$: (0.55, 0.65)	$\mho_{1}$: (0.60, 0.55) $\mho_{2}$: (0.60, 0.52) $\mho_{3}$: (0.68, 0.60)
*V*_3_	$\mho_{1}$: (0.70, 0.40) $\mho_{2}$: (0.65, 0.50) $\mho_{3}$: (0.55, 0.50)	$\mho_{1}$: (0.70, 0.50) $\mho_{2}$: (0.72, 0.55) $\mho_{3}$: (0.68, 0.55)	$\mho_{1}$: (0.64, 0.67) $\mho_{2}$: (0.65, 0.60) $\mho_{3}$: (0.69, 0.58)	$\mho_{1}$: (0.70, 0.69) $\mho_{2}$: (0.62, 0.65) $\mho_{3}$: (0.68, 0.60)	$\mho_{1}$: (0.70, 0.64) $\mho_{2}$: (0.64, 0.58) $\mho_{3}$: (0.62, 0.55)
*V*_4_	$\mho_{1}$: (0.65, 0.50) $\mho_{2}$: (0.60, 0.55) $\mho_{3}$: (0.70, 0.50)	$\mho_{1}$: (0.67, 0.55) $\mho_{2}$: (0.72, 0.58) $\mho_{3}$: (0.68, 0.52)	$\mho_{1}$: (0.70, 0.50) $\mho_{2}$: (0.65, 0.58) $\mho_{3}$: (0.62, 0.55)	$\mho_{1}$: (0.64, 0.60) $\mho_{2}$: (0.70, 0.55) $\mho_{3}$: (0.65, 0.54)	$\mho_{1}$: (0.64, 0.51) $\mho_{2}$: (0.69, 0.65) $\mho_{3}$: (0.61, 0.54)
*V*_5_	$\mho_{1}$: (0.70, 0.60) $\mho_{2}$: (0.65, 0.59) $\mho_{3}$: (0.68, 0.52)	$\mho_{1}$: (0.72, 0.55) $\mho_{2}$: (0.68, 0.50) $\mho_{3}$: (0.65, 0.52)	$\mho_{1}$: (0.70, 0.56) $\mho_{2}$: (0.65, 0.62) $\mho_{3}$: (0.67, 0.60)	$\mho_{1}$: (0.65, 0.60) $\mho_{2}$: (0.66, 0.55) $\mho_{3}$: (0.68, 0.60)	$\mho_{1}$: (0.66, 0.54) $\mho_{2}$: (0.65, 0.56) $\mho_{3}$: (0.63, 0.57)
*V*_6_	$\mho_{1}$: (0.55, 0.72) $\mho_{2}$: (0.53, 0.78) $\mho_{3}$: (0.57, 0.75)	$\mho_{1}$: (0.50, 0.68) $\mho_{2}$: (0.45, 0.72) $\mho_{3}$: (0.40, 0.75)	$\mho_{1}$: (0.50, 0.76) $\mho_{2}$: (0.55, 0.75) $\mho_{3}$: (0.58, 0.70)	$\mho_{1}$: (0.55, 0.72) $\mho_{2}$: (0.63, 0.70) $\mho_{3}$: (0.58, 0.75)	$\mho_{1}$: (0.58, 0.76) $\mho_{2}$: (0.55, 0.72) $\mho_{3}$: (0.60, 0.74)
*V*_7_	$\mho_{1}$: (0.70, 0.62) $\mho_{2}$: (0.69, 0.65) $\mho_{3}$: (0.69, 0.62)	$\mho_{1}$: (0.70, 0.66) $\mho_{2}$: (0.72, 0.63) $\mho_{3}$: (0.74, 0.62)	$\mho_{1}$: (0.70, 0.60) $\mho_{2}$: (0.65, 0.62) $\mho_{3}$: (0.68, 0.66)	$\mho_{1}$: (0.67, 0.55) $\mho_{2}$ (0.68, 0.58) $\mho_{3}$: (0.69, 0.60)	$\mho_{1}$: (0.62, 0.57) $\mho_{2}$: (0.65, 0.54) $\mho_{3}$: (0.62, 0.56)
*V*_8_	$\mho_{1}$: (0.68, 0.55) $\mho_{2}$: (0.70, 0.50) $\mho_{3}$: (0.65, 0.55)	$\mho_{1}$: (0.69, 0.55) ${J}$_2_: (0.72, 0.62) $\mho_{3}$: (0.70, 0.50)	$\mho_{1}$: (0.68, 0.50) $\mho_{2}$: (0.70, 0.55) $\mho_{3}$: (0.72, 0.50)	$\mho_{1}$: (0.68, 0.50) $\mho_{2}$: (0.65, 0.52) $\mho_{3}$: (0.60, 0.56)	$\mho_{1}$: (0.65, 0.58) $\mho_{2}$: (0.60, 0.56) $\mho_{3}$: (0.60, 0.50)
*V*_9_	$\mho_{1}$: (0.58, 0.55) $\mho_{2}$: (0.65, 0.55) $\mho_{3}$: (0.62, 0.60)	$\mho_{1}$: (0.67, 0.59) $\mho_{2}$: (0.73, 0.55) $\mho_{3}$: (0.71, 0.54)	$\mho_{1}$: (0.68, 0.55) $\mho_{2}$: (0.64, 0.50) $\mho_{3}$: (0.69, 0.55)	$\mho_{1}$: (0.68, 0.54) $\mho_{2}$: (0.63, 0.56) $\mho_{3}$: (0.61, 0.52)	$\mho_{1}$: (0.65, 0.55) ${J}$_2_: (0.63, 0.57) $\mho_{3}$: (0.62, 0.55)
*V*_10_	$\mho_{1}$: (0.70, 0.55) $\mho_{2}$: (0.68, 0.55) $\mho_{3}$: (0.60, 0.57)	$\mho_{1}$: (0.68, 0.65) $\mho_{2}$: (0.74, 0.65) $\mho_{3}$: (0.70, 0.62)	$\mho_{1}$: (0.70, 0.68) $\mho_{2}$: (0.65, 0.64) $\mho_{3}$: (0.65, 0.50)	$\mho_{1}$: (0.68, 0.62) $\mho_{2}$: (0.68, 0.60) $\mho_{3}$: (0.64, 0.55)	$\mho_{1}$: (0.62, 0.60) $\mho_{2}$: (0.65, 0.55) $\mho_{3}$: (0.68, 0.60)

*Step 2* Using Eq. (10), DMs’ weights $\psi_{k} :1,2,3$ have been computed as $\left\{ {\psi_{1} = 0.{3765,}\;\psi_{2} = 0.{2875},\;\psi_{3} = 0.{336}0} \right\}$.

*Step 3* Using Eq. (11), the AFF-DM is obtained by considering into rating of the significances of DMs and is depicted in Table 4.

**Table 4:** AFF-DM for HCWDL selection

	*L*_1_	*L*_2_	*L*_3_	*L*_4_	*L*_5_
*V*_1_	(0.465, 0.676)	(0.363, 0.766)	(0.503, 0.687)	(0.446, 0.701)	(0.551, 0.721)
*V*_2_	(0.363, 0.766)	(0.694, 0.539)	(0.640, 0.549)	(0.593, 0.566)	(0.644, 0.557)
*V*_3_	(0.661, 0.460)	(0.717, 0.531)	(0.676, 0.618)	(0.685, 0.647)	(0.673, 0.591)
*V*_4_	(0.668, 0.514)	(0.708, 0.548)	(0.677, 0.539)	(0.681, 0.565)	(0.666, 0.557)
*V*_5_	(0.694, 0.569)	(0.703, 0.525)	(0.690, 0.590)	(0.679, 0.585)	(0.663, 0.556)
*V*_6_	(0.564, 0.747)	(0.468, 0.714)	(0.559, 0.736)	(0.605, 0.724)	(0.592, 0.742)
*V*_7_	(0.710, 0.628)	(0.736, 0.638)	(0.694, 0.625)	(0.696, 0.575)	(0.646, 0.558)
*V*_8_	(0.695, 0.535)	(0.720, 0.551)	(0.716, 0.514)	(0.663, 0.525)	(0.634, 0.546)
*V*_9_	(0.634, 0.566)	(0.721, 0.561)	(0.686, 0.535)	(0.659, 0.539)	(0.650, 0.556)
*V*_10_	(0.682, 0.557)	(0.725, 0.640)	(0.685, 0.603)	(0.684, 0.590)	(0.666, 0.585)

*Step 4* With the help of Table 3 and Eqs. (4), (5) and (12), attributes’ weights are estimated as.

$$\varpi_{j} =$${0.0841, 0.0957, 0.1040, 0.1039, 0.1044, 0.0901, 0.1056, 0.1040, 0.1033, 0.1049}.

*Step 5* Since *V*_1_, *V*_3_ and *V*_6_ are non-beneficial and rest all are of beneficial criteria, thus, the AFF-DM is transformed into normalized AFF-DM. Applying Eq. (13) and Table 4, the normalized AFF-DM is computed and mentioned in Table 5.

**Table 5:** Normalized AFF-decision matrix for HCWDL selection

	*L*_1_	*L*_2_	*L*_3_	*L*_4_	*L*_5_
*V*_1_	(0.676, 0.465)	(0.766, 0.363)	(0.687, 0.503)	(0.701, 0.446)	(0.721, 0.551)
*V*_2_	(0.363, 0.766)	(0.694, 0.539)	(0.640, 0.549)	(0.593, 0.566)	(0.644, 0.557)
*V*_3_	(0.460, 0.661)	(0.531, 0.717)	(0.618, 0.676)	(0.647, 0.685)	(0.591, 0.673)
*V*_4_	(0.668, 0.514)	(0.708, 0.548)	(0.677, 0.539)	(0.681, 0.565)	(0.666, 0.557)
*V*_5_	(0.694, 0.569)	(0.703, 0.525)	(0.690, 0.590)	(0.679, 0.585)	(0.663, 0.556)
*V*_6_	(0.747, 0.564)	(0.714, 0.468)	(0.736, 0.559)	(0.724, 0.605)	(0.742, 0.592)
*V*_7_	(0.710, 0.628)	(0.736, 0.638)	(0.694, 0.625)	(0.696, 0.575)	(0.646, 0.558)
*V*_8_	(0.695, 0.535)	(0.720, 0.551)	(0.716, 0.514)	(0.663, 0.525)	(0.634, 0.546)
*V*_9_	(0.634, 0.566)	(0.721, 0.561)	(0.686, 0.535)	(0.659, 0.539)	(0.650, 0.556)
*V*_10_	(0.682, 0.557)	(0.725, 0.640)	(0.685, 0.603)	(0.684, 0.590)	(0.666, 0.585)

*Step 6–9* Using Table 5 and Eqs. (14) and (15), the measures of WSM and WPM are estimated. After that, with the help of Eq. (16), the WASPAS measure (at $\lambda = 0.5$) is estimated and presented in Table 6. From Table 6, the ranking order of disposal locations for HCW is $L_{2} \succ L_{3} \succ L_{4} \succ L_{5} \succ L_{1} ,$ , therefore, *L*_2_, that is, Bharat Oil and Waste Management Ltd (BOWML) Roorkee is the most desirable HCWDL alternative.

**Table 6:** Degree of WASPAS measure of various HCWDL alternatives

Location options	FF-WSM		FF-WPM		FF-WASPAS	Ranking
	${\mathbb{C}}_{i}^{(1)}$	${\mathbb{S}}\left( {{\mathbb{C}}_{i}^{(1)} } \right)$	${\mathbb{C}}_{i}^{(2)}$	${\mathbb{S}}\left( {{\mathbb{C}}_{i}^{(2)} } \right)$	${\mathbb{C}}_{i} \left( \lambda =0.5 \right)$	
*L*_1_	(0.670, 0.557, 0.807)	0.353	(0.644, 0.582, 0.813)	0.320	0.336	5
*L*_2_	(0.721, 0.536, 0.779)	0.417	(0.720, 0.553, 0.771)	0.413	0.415	1
*L*_3_	(0.690, 0.564, 0.790)	0.374	(0.688, 0.569, 0.788)	0.371	0.373	2
*L*_4_	(0.679, 0.564, 0.798)	0.361	(0.675, 0.571, 0.797)	0.356	0.359	3
*L*_5_	(0.671, 0.565, 0.803)	0.353	(0.668, 0.566, 0.805)	0.349	0.351	4

### Comparative study

In the present section, a comparison is made to confirm the strength of the developed FF- WASPAS framework. To do this, an existing FF-TOPSIS method [44] is considered. The FF-TOPSIS model implicates the given steps:

*Steps 1–5* Similar to the above methodology.

*Step 6* Evaluate the ideal solution (IS) and anti-ideal solution (A-IS), given as.

${\alpha }^{+}$={(0.363, 0.766), (0.694, 0.539), (0.717, 0.531), (0.708, 0.548), (0.703, 0.525), (0.468, 0.714), (0.736, 0.638), (0.716, 0.514), (0.721, 0.561), (0.725, 0.640)} and ${\alpha }^{-}$={(0.551, 0.721), (0.363, 0.766), (0.676, 0.618), (0.666, 0.557), (0.663, 0.556), (0.605, 0.724), (0.646, 0.558), (0.634, 0.546), (0.634, 0.566), (0.666, 0.585)}. Next, the discrimination measures between the options *L*_*i*_ and the IS as well as A-IS on the criterion *V*_*j*_ are determined.

*Step 7* Determine the closeness index *CI* of each option to the IS as

$$ CI\left( {L_{i} } \right) = \frac{{\Upsilon_{i}^{ - } }}{{\Upsilon_{i}^{ + } + \,\Upsilon_{i}^{ - } }}, $$

where $\Upsilon_{i}^{ + } = D\left( {\varepsilon_{ij} ,\,\alpha^{ + } } \right) = \sum\limits_{j = 1}^{n} {\varpi_{j} \sqrt {\frac{1}{2}\left[ {\left( {\left( {t_{ij} } \right)^{3} - \left( {t_{j}^{ + } } \right)^{3} } \right)^{2} + \left( {\left( {f_{ij} } \right)^{3} - \left( {f_{j}^{ + } } \right)^{3} } \right)^{2} + \left( {\left( {\pi_{ij} } \right)^{3} - \left( {\pi_{j}^{ + } } \right)^{3} } \right)^{2} } \right]} }$ and $\Upsilon_{i}^{ - } = D\left( {h_{ij} ,\,\alpha^{ - } } \right) = \sum\limits_{j = 1}^{n} {\varpi_{j} \sqrt {\frac{1}{2}\left[ {\left( {\left( {t_{ij} } \right)^{3} - \left( {t_{j}^{ - } } \right)^{3} } \right)^{2} + \left( {\left( {f_{ij} } \right)^{3} - \left( {f_{j}^{ - } } \right)^{3} } \right)^{2} + \left( {\left( {\pi_{ij} } \right)^{3} - \left( {\pi_{j}^{ - } } \right)^{3} } \right)^{2} } \right]} } .$ Thus, *CI*(*L*_1_) = 0.374, *CI*(*L*_2_) = 0.967, *CI*(*L*_3_) = 0.531, *CI*(*L*_4_) = 0.398 and *CI*(*L*_5_) = 0.244.

*Step 8* Rank the HCWDL options as $L_{2} \succ L_{3} \succ L_{4} \succ L_{1} \succ L_{5} ,$ that is, the most effective option for HCWDL is Bharat Oil and Waste Management Ltd (BOWML) Roorkee (*L*_2_).

**Fig. 3:**
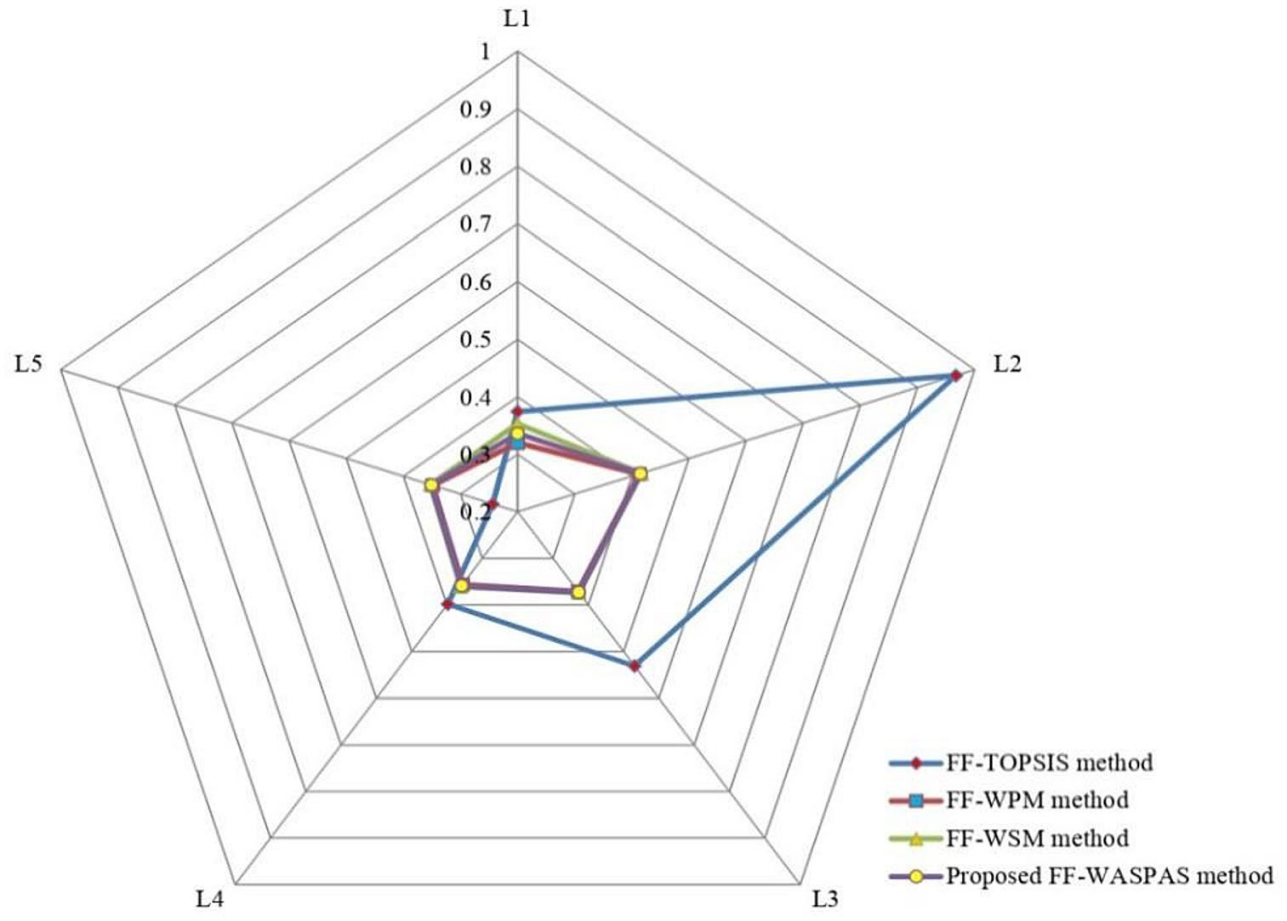
Variation of degree of significance of each HCWDL alternatives

**Fig. 4:**
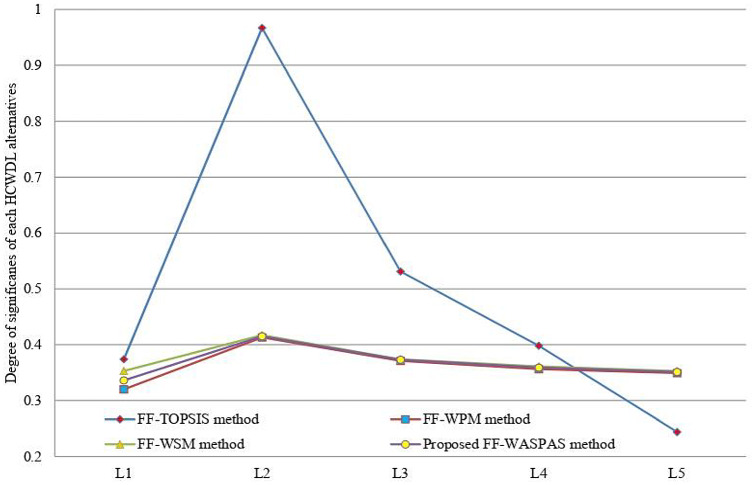
Comparison of degree of significance of each HCWDL alternatives with various methods

Here, a comparison is discussed with some existing approaches, comprising FF-TOPSIS method [44], FF-WPM method [45] and IRN-BWM-Dombi-Bonferroni method [56]. From FF-TOPSIS model, the preference order of the HCWDL options is $L_{2} \succ L_{3} \succ L_{4} \succ L_{1} \succ L_{5} ,$ and the most suitable HCWDL alternative is Bharat Oil and Waste Management Ltd (BOWML) Roorkee (*L*_2_). Next, by comparing with FF-WPM method, the ranking order of the HCWDL alternatives is $L_{2} \succ L_{3} \succ L_{4} \succ L_{5} \succ L_{1} ,$ and the most suitable HCWDL alternative is *L*_2_. From IRN-BWM-Dombi-Bonferroni [56] method, the final ranking of the HCWDL alternative is $L_{2} \succ L_{1} \succ L_{2} \succ L_{4} \succ L_{5} ,$ and the most suitable HCWDL alternative is Bharat Oil and Waste Management Ltd (BOWML) Roorkee (*L*_2_). Hence, it has been concluded that the most suitable HCWDL alternative (*L*_2_) is similar by all approaches, whereas the ranking order results are slightly vary with various models. Figures 3 and 4 are depicted the variation of the degree of significances of each HCWDL alternatives with various approaches.

**Fig. 5:**
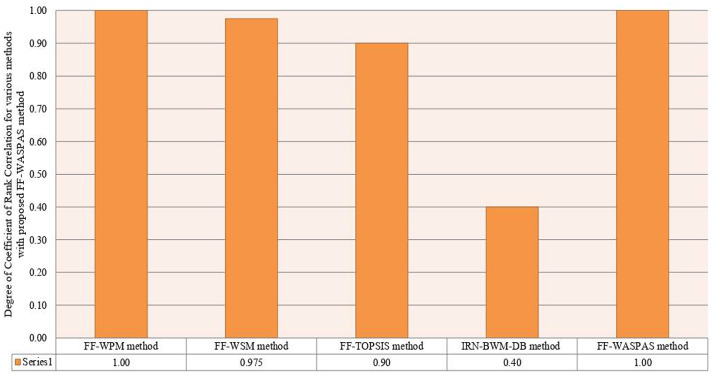
Correlation design of different measures of WASPAS model with other models

From Fig. 5, it is observed that the introduced methodology is highly stable with existing models. To preserve the consistency in the methodology-related comparison, the models given by [44, 45] and Yazdani et al. [56] are considered. From Fig. 5, it is clear that the dependability of the introduced framework is high with existing methods. The Spearman correlation values of the proposed method (WASPAS measure, *λ* = 0.5), FF-TOPSIS, WSM (*λ* = 1.0) measure, WPM (*λ* = 0.0) and IRN-BWM-Dombi-Bonferroni method with WASPAS measure solution are given by (1.00, 0.90, 0.975, 1.00, 0.40). Generally, the outcomes of the FF-WASPAS framework are discussed as follows:

- The WASPAS approach, a utility scoring model for MCDM, selects an option which has the maximum score (or the utmost utility), whereas the previous approaches, which are compromising degree procedures, prefer an option which is nearest to the ideal solution.
- WASPAS is a combination of the WSM and WPM. The accuracy of the WASPAS approach is more consistent than WSM and WPM. This method facilitates us to achieve the maximum accuracy of assessment, utilizing the introduced framework for optimization of weighted aggregated mapping.
- In the proposed model, the criteria weights are computed-based on proposed entropy and score function and are given in FFNs, whereas in Yazdani et al. [56], the criteria weights are estimated based on BWM and are mentioned in terms of IRNs. On the other hand, FF-TOPSIS and FF-WPM models, the criteria weights are assumed, which leaves no room for managing the ambiguity.
- The proposed framework could offer a more precise description under an uncertain environment because of evaluating the criteria and DMs’ weights and utilizing them in the process of the proposed method. In addition, two other considered as central features in the procedure of this method lead the computational results to a reliable solution. These features comprise the last aggregation method to evade the loss of data and to tailor the introduced framework based on FFSs information.

### Implementation and discussions

This study has been developed to assess the disposal of HCWs from different locations of Uttarakhand (India) with uncertain information.

In this study, an integrated FF-WASPAS framework has proposed and then employed to select the most appropriate location for HCW disposal in Uttarakhand (India). An illustrative case study has presented to demonstrate some significant insights regarding considered criteria and chosen five disposal locations. The obtained outcomes by the introduced methodology show that the option *L*_2_, i.e., Bharat Oil and Waste Management Ltd (BOWML), Roorkee is the most suitable location for the present case study. Furthermore, a comparison with existing approaches has also been discussed to reveal the robustness of the proposed framework. And thus, *L*_2_ is the most appropriate location among others by different methods. Consequently, the developed method has significant information that can be used by the healthcare experts or managers in taking strategic or operational decisions in HCWDLs evaluation.

Here, it has been observed that the factors *V*_7_ (Possibility of future development), *V*_10_ (Level of satisfaction among residents to the location selection) and *V*_5_ (the prevailing environmental friendly services (air, water, energy, and electricity supply) are the top three criteria. All these factors indirectly or directly have an influence in HCWDL selection. Whereas, the criteria *V*_3_ (distance to a complex of waste sorting), *V*_8_ (availability of employees), *V*_4_ (geographic and geologic circumstances) and *V*_9_ (sensitivity towards environment, local and territorial rules or protocols) has medium to slightly high significance. In contrast, the criteria *V*_1_ (potential risk of intrusion and emission), *V*_2_ (distance to the urban and city infrastructure and society), and *V*_6_ [land price (in m^2^) and other costs (transportation and maintenance) in the specific zone] have been observed as the least important factors.

Without loss of generality, the introduced method would be correspondingly suitable to other realistic concerns. Also, the managers/experts can use the proposed model to study the relationships among economic, environmental, and social aspects, and utilize the outcomes to influence and assure stronger law and strategy execution on sustainability. In addition, their policy implications and recommendations to enhance adherence to HCWT rules are conferred below.

- The government hospitals (GHs) require solid support skills to protect adequate funding from the state administration to start utilizing the facilities of common waste management facilities (CWMFs). The state health structures development project can show a key impact in the application of bio-medical waste (BMW) guidelines by the GHs.
- The pollution control board (PCB) also wants to performance as a facilitator between the GHs and the CWMFs more efficiently so as to fast agreement can be touched between the two systems.
- Diverse kinds of posters are being utilized by two CMFWs in the state. These can origin misperceptions among hospital employees, specifically among those who reformed their CMFW. The PCB should certify that diverse messages are not conversed by the CMFWs to the hospitals.
- The non-use of uniform color bins may source misperception among the lower-level employee, which may consequence in preventable mix-ups of segregated waste. Therefore, the PCB and CWMFs should assert that the hospitals and other systems follow to uniform color coding for both bins and plastic materials that they use.

## Conclusions

The proper management of HCW is a public concern, which involves effective planning and procedure. The HCWDL selection is a crucial portion of municipal solid waste management. In this paper, a collective MCDM methodology, named as FF-WASPAS, is established by combining the classical WASPAS method, score function, and entropy measure within FFSs context. In the proposed framework, the score function and entropy measure-based procedure has been utilized to assess the criteria weights for HCWDL selection. For this, novel score function and entropy measure have been introduced under FFSs context. Next, the proposed framework has been used on an empirical study of HCWDL selection in Uttarakhand, India, which proves the effectiveness and practicability of the FF-WASPAS approach. From the sustainability perspectives, a comprehensive evaluation index system has been built for this case study, which consists of three main criteria: environmental, economic, and social. Further, a comparative study has been carried out to validate the outcomes. Next, sensitivity analysis has been discussed to analyze the assessment and selection process of the locations and also, verify the robustness of the developed approach. The results of the analysis prove that the introduced FF-WASPAS model is more effective, reliable, and stable and has less computational complexity than existing approaches within FFSs environment. In addition, it provides a new weight-determining procedure to assess more accurate criteria weights that improves the permanence of the introduced model. This study provides unique theoretical as well as practical contributions in the context of HCWDL selection with uncertainty. Thus, the proposed method can be employed by health experts or organizations to assess and choose the locations in a most efficient and flexible way.

The limitation of the proposed method is as (1) all criteria are assumed to be independent. In fact, there are interrelationships among criteria in practical decision-making problems, and (2) the assessment index system should include more sustainable dimensions of the criteria.

This study provides directions for future research. First, the developed framework can be employed in different decision-making applications in other industries. Second, it is suggested to develop new procedures to compute the mutual interactions between the criteria. Third, it is interesting and important to develop different types of aggregation operators and information measures (entropy, similarity, distance, and divergence measures) for FFSs and interval-valued FFSs. Last but not least, the future study can develop new methods like DNMA, GLDS, ORESTE, MARCOS, and others under Fermatean fuzzy and interval-valued Fermatean fuzzy environment and employ to choose HCWDL selection problem for different regions or different countries.

## References

[1] Agarwal S, Kant R, Shankar R (2020). Evaluating solutions to overcome humanitarian supply chain management barriers: a hybrid fuzzy SWARA—fuzzy WASPAS approach. Int J Disaster Risk Reduct.

[2] Ali J, Bashir Z, Rashid T (2021). WASPAS-based decision making methodology with unknown weight information under uncertain evaluations. Expert Syst Appl.

[3] Almeida C, Bonilla S, Giannetti B (2013). Cleaner production initiatives and challenges for a sustainable world: an introduction to this special volume. J Clean Prod.

[4] Alumur S, Kara BY (2007). A new model for the hazardous waste location-routing problem. Comput Oper Res.

[5] Amma BB, Melliani S, Chadli LS (2019). The existence and uniqueness of intuitionistic fuzzy solutions for intuitionistic fuzzy partial functional differential equations. Int J Differ Equ.

[6] Andrinopoulos K, Kerrigan D, Ellen JM, Ellen M (2016). Understanding sex partner selection from the perspective of inner‐city black adolescents. Perspect Sex Reprod Health.

[7] Arıkan E, Şimşit-Kalender ZT, Vayvay Ö (2017). Solid waste disposal methodology selection using multi-criteria decision making methods and an application in Turkey. J Clean Prod.

[8] Atanassov KT (1986). Intuitionistic fuzzy sets. Fuzzy Sets Syst.

[9] Aydemir SB, Gunduz SY (2020). Fermatean fuzzy TOPSIS method with dombi aggregation operators and its application in multi-criteria decision making. J Intell Fuzzy Syst.

[10] Boran FE, Genç S, Kurt M, Akay D (2009). A multi-criteria intuitionistic fuzzy group decision making for supplier selection with TOPSIS method. Expert Syst Appl.

[11] Chauhan A, Singh A (2016). Healthcare waste management: a state-of-the-art literature review. Int J Environ Waste Manag.

[12] Chauhan A, Singh A (2016). A hybrid multi-criteria decision making method approach for selecting a sustainable location of healthcare waste disposal facility. J Clean Prod.

[13] Deveci M, Canıtez F, Gökaşar I (2018). WASPAS and TOPSIS based interval type-2 fuzzy MCDM method for a selection of a car sharing station. Sustain Urban Areas.

[14] Ekmekçioğlu M, Kaya T, Kahraman C (2010). Fuzzy multi criteria disposal method and site selection for municipal solid waste. Waste Manag.

[15] Erkut E, Karagiannidis A, Perkoulidis G, Tjandra SA (2008). A multicriteria facility location model for municipal solid waste management in North Greece. Eur J Oper Res.

[16] Ertugrul I, Karakasoglu N (2008). Comparison of fuzzy AHP and fuzzy TOPSIS methods for facility location selection. Int J Adv Manuf Technol.

[17] Eskandari M, Homaee M, Falamaki A (2016). Landfill site selection for municipal solid wastes in mountainous areas with landslide susceptibility. Environ Sci Pollut Res.

[18] Gergin Z, Tunçbilek N, Esnaf Ş (2019). Clustering approach using artificial bee colony algorithm for healthcare waste disposal facility location problem. Int J Oper Res Inf Syst.

[19] Ghorabaee MK, Zavadskas EK, Amiri M, Esmaeili A (2016). Multi-criteria evaluation of green suppliers using an extended WASPAS method with interval type-2 fuzzy sets. J Clean Prod.

[20] Gorsevski PV, Donevska KR, Mitrovski CD, Frizado JP (2012). Integrating multi-criteria evaluation techniques with geographic information systems for landfill site selection: a case study using ordered weighted average. Waste Manag.

[21] Hariz HA, Dönmez CÇ, Sennaroglu B (2017). Siting of a central healthcare waste incinerator using GIS-based multi-criteria decision analysis. J Clean Prod.

[22] He X, Li Y, Qin K, Meng D (2020). Distance measures on intuitionistic fuzzy sets based on intuitionistic fuzzy dissimilarity functions. Soft Comput.

[23] Hu J, Yang Y, Zhang X, Chen X (2018). Similarity and entropy measures for hesitant fuzzy sets. Int Trans Oper Res.

[24] IndiaStat (2013). http://www.indiastat.com/table/environmentandpollution/11/solidwaste/261/910950/data.aspx. Accessed 06 Feb 2016

[25] Kahraman C, Ghorabaee MK, Zavadskas EK, CevikOnar S, Yazdani M, Oztaysi B (2017). Intuitionistic fuzzy EDAS method: an application to solid waste disposal site selection. J Environ Eng Landsc Manag.

[26] Karamouz M, Zahraie B, Kerachian R, Jaafarzadeh N, Mahjouri N (2007). Developing a master plan for hospital solid waste management: a case study. Waste Manag.

[27] Khan D, Samadder SR (2015). A simplified multi-criteria evaluation model for landfill site ranking and selection based on AHP and GIS. J Environ Eng Landsc Manag.

[28] Mardani A, Saraji MK, Mishra AR, Rani P (2020). A novel extended approach under hesitant fuzzy sets to design a framework for assessing the key challenges of digital health interventions adoption during the COVID-19 outbreak. Appl Soft Comput.

[29] Melo MT, Nickel S, Saldanha-da-Gama F (2009). Facility location and supply chain management e a review. Eur J Oper Res.

[30] Mishra AR, Jain D, Hooda DS (2016). On logarithmic fuzzy measures of information and discrimination. J Inf Optim Sci.

[31] Mishra AR, Mardani A, Rani P, Zavadskas EK (2020). A novel EDAS approach on intuitionistic fuzzy set for assessment of health-care waste disposal technology using new parametric divergence measures. J Clean Prod.

[32] Mishra AR, Rani P, Pandey K (2021). Fermatean fuzzy CRITIC-EDAS approach for the selection of sustainable third-party reverse logistics providers using improved generalized score function. J Ambient Intell Humaniz Comput.

[33] Mishra AR, Rani P (2018). Interval-valued intuitionistic fuzzy WASPAS method: application in reservoir flood control management policy. Group Decis Negot.

[34] Mishra AR, Rani P, Pardasani KR, Mardani A (2019). A novel hesitant fuzzy WASPAS method for assessment of green supplier problem based on exponential information measures. J Clean Prod.

[35] Mohagheghi V, Mousavi SM (2020). D-WASPAS: addressing social cognition in uncertain decision-making with an application to a sustainable project portfolio problem. Cogn Comput.

[36] Mokhtarian MN, Sadi-Nezhad S, Makui A (2014). A new flexible and reliable interval valued fuzzy VIKOR method based on uncertainty risk reduction in decision making process: an application for determining a suitable location for digging some pits for municipal wet waste landfill. Comput Ind Eng.

[37] Nema AK, Gupta SK (1999). Optimization of regional hazardous waste management systems: an improved formulation. Waste Manag.

[38] Nolz PC, Nationale E, Charpak CMPG, Gardanne F, Absi N, Feillet D (2014). A stochastic inventory routing problem for infectious medical waste collection. Networks.

[39] Rabbani M, Heidari R, Farrokhi-Asl H, Rahimi N (2018). Using metaheuristic algorithms to solve a multi-objective industrial hazardous waste location-routing problem considering incompatible waste types. J Clean Prod.

[40] Rakas J, Teodorović D, Kim T (2004). Multi-objective modeling for determining location of undesirable facilities. Transp Res Part D Transp Environ.

[41] Rani P, Mishra AR (2020). Multi-criteria weighted aggregated sum product assessment framework for fuel technology selection using q-rung orthopair fuzzy sets. Sustain Prod Consum.

[42] Schitea D, Deveci M, Iordache M, Bilgili K, Akyurt İZ, Iordache I (2019). Hydrogen mobility roll-up site selection using intuitionistic fuzzy sets based WASPAS, COPRAS and EDAS. Int J Hydrogen Energy.

[43] Rani P, Mishra AR, Pardasani KR (2020). A novel WASPAS approach for multi criteria physician selection problem with intuitionistic fuzzy type-2 sets. Soft Comput.

[44] Senapati T, Yager RR (2019). Fermatean fuzzy sets. J Ambient Intell Humaniz Comput.

[45] Senapati T, Yager RR (2019). Some new operations over Fermatean fuzzy numbers and application of Fermatean fuzzy WPM in multiple criteria decision making. Informatica.

[46] Senapati T, Yager RR (2019). Fermatean fuzzy weighted averaging/geometric operators and its application in multi-criteria decision-making methods. Eng Appl Artif Intell.

[47] Şener Ş, Sener E, Karagüzel R (2011). Solid waste disposal site selection with GIS and AHP methodology: a case study in Senirkent-Uluborlu (Isparta) Basin, Turkey. Environ Monit Assess.

[48] Shahzadi G, Akram M, Al-Kenani AN (2020). Decision-making approach under Pythagorean fuzzy yager weighted operators. Symmetry.

[49] Shwesin AT, Luan S, Xu Q (2019). Application of multi-criteria decision approach for the analysis of medical waste management systems in Myanmar. J Clean Prod.

[50] Thakur V, Ramesh A (2017). Healthcare waste disposal strategy selection using grey-AHP approach. Benchmark Int J.

[51] Thao NX, Smarandache F (2019). A new fuzzy entropy on Pythagorean fuzzy sets. J Intell Fuzzy Syst.

[52] Turskis Z, Zavadskas EK, Antucheviciene J, Kosareva N (2015). A hybrid model based on fuzzy AHP and fuzzy WASPAS for construction site selection. Int J Comput Commun Control.

[53] Wichapa N, Khokhajaikiat P (2017). Solving multi-objective facility location problem using the fuzzy analytical hierarchy process and goal programming: a case study on infectious waste disposal centers. Oper Res Perspect.

[54] Windfeld ES, Brooks MSL (2015). Medical waste management—a review. J Environ Manag.

[55] Yager RR (2014). Pythagorean membership grades in multicriteria decision making. IEEE Trans Fuzzy Syst.

[56] Yazdani M, Tavana M, Pamučar D, Chatterjee P (2020). A rough based multi-criteria evaluation method for healthcare waste disposal location decisions. Comput Ind Eng.

[57] Zadeh LA (1965). Fuzzy sets. Inf Control.

[58] Zavadskas EK, Baušys R, Lazauskas M (2015). Sustainable assessment of alternative sites for the construction of a waste incineration plant by applying WASPAS method with single-valued neutrosophic set. Sustainability.

[59] Zavadskas EK, Turskis Z, Antucheviciene J, Zakarevicius A (2012). Optimization of weighted aggregated sum product assessment. Electron Electr Eng.

[60] Zheng Y, Xu J, Chen H (2020). TOPSIS-based entropy measure for intuitionistic trapezoidal fuzzy sets and application to multi-attribute decision making. Math Biosci Eng.

